# The endocannabinoid system as a putative target for the development of novel drugs for the treatment of psychiatric illnesses

**DOI:** 10.1017/S0033291723002465

**Published:** 2023-11

**Authors:** Matthew N. Hill, Margaret Haney, Cecilia J. Hillard, Debra S. Karhson, Haley A. Vecchiarelli

**Affiliations:** 1 Departments of Cell Biology and Anatomy & Psychiatry, Cumming School of Medicine, Hotchkiss Brain Institute and The Mathison Centre for Mental Health Research and Education, University of Calgary, Calgary, Canada; 2 Department of Psychiatry, New York State Psychiatric Institute and Columbia University Irving Medical Center, New York, USA; 3 Department of Pharmacology and Toxicology, Neuroscience Research Center, Medical College of Wisconsin, Milwaukee, USA; 4 Department of Psychology, University of New Orleans, New Orleans, USA; 5 Division of Medical Sciences, University of Victoria, Victoria, Canada

**Keywords:** Anxiety, autism, cannabis, CB1, endocannabinoid, FAAH, MAGL, psychiatric illness, PTSD, substance use disorder

## Abstract

Cannabis is well established to impact affective states, emotion and perceptual processing, primarily through its interactions with the endocannabinoid system. While cannabis use is quite prevalent in many individuals afflicted with psychiatric illnesses, there is considerable controversy as to whether cannabis may worsen these conditions or provide some form of therapeutic benefit. The development of pharmacological agents which interact with components of the endocannabinoid system in more localized and discrete ways then via phytocannabinoids found in cannabis, has allowed the investigation if direct targeting of the endocannabinoid system itself may represent a novel approach to treat psychiatric illness without the potential untoward side effects associated with cannabis. Herein we review the current body of literature regarding the various pharmacological tools that have been developed to target the endocannabinoid system, their impact in preclinical models of psychiatric illness and the recent data emerging of their utilization in clinical trials for psychiatric illnesses, with a specific focus on substance use disorders, trauma-related disorders, and autism. We highlight several candidate drugs which target endocannabinoid function, particularly inhibitors of endocannabinoid metabolism or modulators of cannabinoid receptor signaling, which have emerged as potential candidates for the treatment of psychiatric conditions, particularly substance use disorder, anxiety and trauma-related disorders and autism spectrum disorders. Although there needs to be ongoing clinical work to establish the potential utility of endocannabinoid-based drugs for the treatment of psychiatric illnesses, the current data available is quite promising and shows indications of several potential candidate diseases which may benefit from this approach.

Cannabis has been widely used for centuries around the world for both recreational and medicinal purposes (Mechoulam & Parker, 2013). Many of the putative medical uses of cannabis often relate to psychiatric conditions, particularly those associated with anxiety, hyperarousal, stress regulation and mood (Mechoulam & Parker, 2013). While cannabis remains illegal in most countries in the world today, there is a strong advocacy and patient-oriented network that purports the medical benefit of this plant. While studying cannabis has been surprisingly difficult in the modern era, various forms of research both support the idea that cannabis may have benefits for some psychiatric conditions and that cannabis use is strongly associated with adverse outcomes and negative disease trajectories, creating a complicated picture regarding its associated potential therapeutic benefits. While cannabis contains dozens, and possibly hundreds, of unique phytocannabinoids, Δ^9^-tetrahydrocannabinol (THC) was isolated and confirmed as the primary psychoactive constituent of cannabis almost 60 years ago (Gaoni & Mechoulam, 1964). The isolation and identification of THC have allowed the field to move forward significantly in understanding the biological mechanisms of cannabis, most importantly through triggering the discovery of the endocannabinoid (eCB) system. The eCB system is composed of eCB molecules themselves, the enzymes which generate and inactivate the eCBs and the receptors through which eCBs and THC exert their effects on physiology (Hillard, 2015). The discovery of the eCB system was a hugely significant advance in the field of cannabinoid pharmacology as it provided both a molecular target for THC and allowed for further exploration of the biological roles and therapeutic potential of harnessing this system.

While many advocacy and patient-oriented groups have argued for the use of whole cannabis as a therapeutic approach, there are several key issues that should be addressed. First, cannabis is a complex plant with varying chemical compositions across different strains or chemovars, most of which have largely unknown biological actions. More so, the interactions that could occur through the co-exposure to these molecules represent an unknown that makes the medical community generally wary of this approach. Typical medications come in standardized dosing and formulations for consistency across time and patients, and currently this is virtually impossible to do with cannabis. Second, approximately 19% of individuals who use cannabis develop a cannabis use disorder (CUD) (Hasin, 2018) and therefore this risk must be factored into any therapeutic approach when considering the benefit *v.* the potential harms. To date, the few studies that have looked at therapeutic effect of cannabis itself have not found robust outcomes associated with cannabis, and as such the overwhelming view is that the potential harms associated with cannabis itself may likely outweigh its potential to provide therapeutic benefit. Third, chronic use of cannabis is known to be associated with alteration of components of the eCB system (Ceccarini et al., 2015). This indicates that there is potential for excess use of cannabis to compromise normative eCB function, which in turn could ultimately make a disease process worse if the pathology of that disease already involves dysfunction of the eCB system. Fourth, cannabis use exhibits clear relationships with either the development or worsening of psychiatric conditions (De Aquino et al., 2018), particularly schizophrenia and possibly depression (Sideli, Quigley, La Cascia, & Murray, 2020). While the nature of these relationships is still not entirely clear (Hill, 2015), these risks contribute to the potential harms of utilizing cannabis itself as a therapeutic approach. Thus, the notion that cannabis will become a therapeutic tool that is accepted by the broad medical community seems highly unlikely given the adverse effects and risks for harm described above. Contrastingly, there is a wide-spread recognition that there is a biological basis by which cannabis benefits many individuals, which has led to the perspective that there may be differential ways to target the eCB system independent of cannabis, which could still harness the therapeutic potential of the eCB system, without the potential risks and harms associated with cannabis itself. As such, the aim of this review is to provide an up-to-date discussion of pharmacological approaches targeting the eCB system and the current state of knowledge of how these approaches are being tested for certain psychiatric conditions, with a focus on substance use disorders, trauma related disorders, and autism.

## The endocannabinoid system and its pharmacology

The eCB system is involved in the regulation of many important brain functions, including emotional responses, control of posture and movement, learning and memory, inflammation, drive to eat and sleep, and sensation of pain (Lutz, 2020). The most well-studied components of the brain eCB system include the cannabinoid receptors, CB1R and CB2R; their endogenous ligands, the eCBs, *N*-arachidonoylethanolamine (anandamide; AEA) and 2-arachidonolyglycerol (2-AG); and the enzymes and transporters that regulate free eCB concentrations (Hillard, 2015).

Discovery that eCBs are both derivatives of arachidonic acid and highly lipophilic was in accord with the high lipophilicity of THC (Leuschner, Harvey, Bullingham, & Paton, 1986) and furthered the appreciation of the wide array of chemicals that serve as ligands for G protein-coupled receptors (GPCRs). The identification of the eCB system and the elucidation of its role in the regulation of synaptic transmission has been transformative to our understanding of brain neurochemistry. For example, identification of the cannabinoid receptors provided the long-sought mechanism for the behavioral, physiological and immune effects of THC, that were uncovered in the 1970's and 1980's using classical pharmacological methods (Dewey, 1986). Additionally, identification of the synaptic impact of eCB signaling provided a molecular mechanism for the process of activity-dependent, retrograde regulation of synaptic activity that was functionally characterized in the 1980's (Pitler & Alger, 1994). Furthermore, the eCB system provided a molecular mechanism by which brain glucocorticoids (Di, Malcher-Lopes, Halmos, & Tasker, 2003; Hill, Karatsoreos, Hillard, & McEwen, 2010) and estrogens (Huang & Woolley, 2012) produce rapid, nongenomic effects in the brain.

A defining feature of the brain eCB system is its involvement in retrograde regulation of neurotransmitter release (Freund, Katona, & Piomelli, 2003). CB1Rs are GPCRs present at high density on axon terminals (Tsou, Brown, Sañudo-Peña, Mackie, & Walker, 1998). When CB1Rs are activated by an agonist, G protein-mediated signaling cascades are initiated that result in inhibition of the opening of voltage-gated calcium channels (Mackie & Hille, 1992; Nogueron, Porgilsson, Schneider, Stucky, & Hillard, 2001) and membrane hyperpolarization due to increased opening of potassium channels (Mu, Zhuang, Kirby, Hampson, & Deadwyler, 1999). These changes result in reduced probability of synaptic vesicle engagement with the axonal membrane and, therefore, reduced neurotransmitter release. CB1Rs are expressed on axonal terminals of several types of neurons, including cortical glutamatergic projection neurons, GABAergic interneurons, medium spiny neurons, and serotonergic neurons (Hillard, 2015). As a result, CB1R activation can inhibit excitatory, inhibitory, and modulatory neurotransmission at a large number of synapses. In addition to these canonical actions of eCB molecules, there is also a rapidly developing appreciation that eCBs may alter neurotransmission through non-canonical mechanisms, including effects on astrocytes and direct actions on mitochondria.

### CB1R pharmacology: orthosteric ligands

The phytocannabinoid, Δ^9^-THC, binds to the CB1R with *K*_I_ values in the 25–75 nM range (Devane, Dysarz, Johnson, Melvin, & Howlett, 1988; Kearn, Greenberg, DiCamelli, Kurzawa, & Hillard, 1999) and exhibits incomplete efficacy to induce G protein activation, thus is a partial agonist (Burkey, Quock, Consroe, Roeske, & Yamamura, 1997; Dutta, Selvam, Das, & Shukla, 2022). Other plant-derived cannabinoids with agonist activity include Δ^8^-THC, which is pharmacologically indistinguishable from Δ^9^-THC (Martin et al., 1993), and Δ^9^-tetrahydrocannabiphorol, a THC analog with a seven carbon alkyl side chain in place of the pentyl side chain of THC (Citti et al., 2019). Interestingly, another phytocannabinoid, Δ^9^-tetrahydrocannabivarin (THCV), in which the pentyl is replaced with a propyl side chain, also binds with nanomolar affinity but is an antagonist of the CB1R (Thomas et al., 2005). 11-Hydroxy-Δ^9^-THC, formed from Δ^9^-THC *in vivo* by the hepatic enzymes cytochrome P450 (CYP)2C9 (primary) and CYP2D6 and CYP2C19 (minor) (Patilea-Vrana, Anoshchenko, & Unadkat, 2019), has higher affinity for the CB1R (Kearn et al., 1999) and is at least as active as Δ^9^-THC as a CB1R agonist (Wiley, Barrus, Farquhar, Lefever, & Gamage, 2021).

Structure-activity studies using the structure of THC as a starting point have identified many additional CB1R agonists, including some with extremely high potency and efficacy in behavioral and physiological assays (Domino, Hardman, & Seevers, 1971; Hardman, Domino, & Seevers, 1971). Levonantradol, developed by scientists at Pfizer (Johnson et al., 1981), was studied in clinical trials for its anti-emetic properties (Diasio, Ettinger, & Satterwhite, 1981). Further modification of the nantradol structure led to the CP family of compounds, including CP55940, that have very high affinity and efficacy and are used in preclinical studies. A completely different structural class of high affinity and high efficacy CB1R agonists, the aminoalkylindoles, was identified and expanded by scientists at Sterling Winthrop (D'Ambra et al., 1992). Unlike the levonantradol/CP series of compounds, WIN 55212-2 is not structurally related to THC and, therefore, not subject to governmental regulations that control access to analogs of THC for research purposes.

High affinity, high efficacy CB1R agonists that are relatively easy to synthesize have been obtained and used by humans outside of medical advice for recreational and medicinal purposes (Coronado-Álvarez et al., 2021). These synthetic cannabinoid agonists can have serious adverse effects and have been associated with a wide array of severe psychiatric, cardiovascular, and gastrointestinal consequences (Alipour, Patel, Shabbir, & Gabrielson, 2019; Courts, Maskill, Gray, & Glue, 2016) and lethality (Giorgetti, Busardò, Tittarelli, Auwärter, & Giorgetti, 2020). This strongly indicates that there is little reason to explore high efficacy CB1R agonists as therapeutic agents.

Currently available direct CB1R agonists, all of which are relatively low efficacy agonists, approved by the US FDA for use in humans include the orally administered compounds nabilone (brand name Cesamet) and dronabinol (synthetic Δ^9^-THC, brand name Marinol). Nabiximol (brand name Sativex), a 1:1 combination of Δ^9^-THC and cannabidiol (CBD) extracted from the cannabis plant administered via oromucosal spray, is approved for use in Europe and Canada.

Orthosteric antagonists of the CB1R have been identified. Rimonabant (brand name Acomplia), is a high affinity, brain penetrant, inverse agonist that was approved in Europe for a short time to treat symptoms of metabolic disorder and contribute to the cessation of smoking (Gelfand & Cannon, 2006). It was removed from the market over concerns of psychiatric adverse effects, which included anxiety, insomnia and increased suicidality (Christensen, Kristensen, Bartels, Bliddal, & Astrup, 2007). This adverse effect profile was predictable based upon what was known even then about the role of the brain eCB system to regulate hedonia and affective states (Hill & Gorzalka, 2009; van der Stelt & Di Marzo, 2003). Newer CB1R antagonists that are unable to enter the brain have been developed with the hypothesis that this drug class will be useful for the treatment of metabolic disorder and will avoid CNS-related adverse effects (Nguyen, Thomas, & Zhang, 2019).

### CB1R pharmacology: allosteric modulators

Orthosteric agonists, such as THC, bind to the same site of the receptor as the eCBs and can induce signaling at CB1Rs. On the other hand, allosteric ligands (also called allosteric modulators) bind to other regions of the receptor and modulate orthosteric ligand binding and/or signaling. Positive allosteric modulators (PAMs) enhance orthosteric agonist signaling, while negative allosteric modulators (NAMs) inhibit agonist signaling. Thus, PAMs can activate the eCB system, but will do so selectively by potentiating CB1R activity only at receptors with orthosterically bound eCBs, while NAMs will dampen CB1R activity driven by orthosteric CB1R agonists.

Several CB1R PAMs have been identified and characterized. 6-Methyl-3-[2-nitro-1-(thiophen-2-yl)ethyl]-2-phenyl-1H-indole (ZCZ011), increases binding of orthosteric agonists and enhances CB1R signaling (Ignatowska-Jankowska et al., 2015). ZCZ011 has been shown to alleviate the effects of THC withdrawal (Trexler, Eckard, & Kinsey, 2019). Another indole, 3-(2-nitro-1-phenylethyl)-2-phenyl-1H-indole (GAT211), exhibits both PAM and allosteric agonist activity (i.e. activation of signaling in the absence of an orthosteric agonist) in multiple assays (Laprairie et al., 2017). GAT211 is a racemic mixture of a CB1R allosteric agonist, GAT228, and a CB1R PAM, GAT229, and the dual modulatory effects of the racemic GAT211 likely result from separate effects of the two molecules (Laprairie et al., 2017). GAT211 has been shown to mimic the effects of CB1R agonists to reduce pain, symptoms of psychosis, and symptoms of Huntington's Disease (Garai et al., 2021; Laprairie et al., 2019; McElroy et al., 2021; Slivicki et al., 2018).

NAMs have also had a fair amount of preclinical development, but a serendipitous finding that the steroid hormone pregnenolone exhibits signaling-specific inhibitory effects at the CB1R (Vallée et al., 2014) has driven renewed interest in this potential class of drug as the toxicology and biology of pregnenolone is already established. While synthetic and specific NAMs for the CB1R have not seen significant clinical development, analogs of pregnenolone have been developed, which maintain its ability to selectively inhibit discrete signaling cascades activated by CB1Rs, and are moving through human trials.

### CB1R indirect agonism via increased endocannabinoid concentrations

Endocannabinoid availability to CB1Rs is in large part regulated by the summation of their synthesis and degradation (Hillard, 2015). Synthesis of 2-AG is induced by receptors that activate phospholipase C and requires diacylglycerol lipase (DAGL). Mechanisms regulating AEA synthesis are not as well understood, but likely include both constitutive and evoked synthesis. Neither eCB are stored in vesicles and increased free concentrations are associated with increased CB1R activation. Hydrolytic cleavage of the ester and amide bonds of 2-AG and AEA, respectively, results in conversion of the eCBs into free arachidonic acid and is the primary mechanism for their inactivation in brain.

### Inactivation of 2-AG

Monoacylglycerol lipase (MAGL), responsible for approximately 80% of 2-AG hydrolysis to arachidonic acid in brain (Blankman, Simon, & Cravatt, 2007), is enriched in axon terminals (Gulyas et al., 2004). MAGL is constitutively active and also present in astrocytes (Viader et al., 2015), microglia (Kouchi, 2015), and oligodendrocytes (Moreno-Luna et al., 2021). Irreversible MAGL inhibitors including JZL184 (Long et al., 2009) and MJN110 (Feja et al., 2020), and reversible inhibitors based upon a diphenylsulfide-benzoylpiperidine scaffold (Bononi et al., 2021; Granchi et al., 2021) have been developed.

Preclinical studies have demonstrated that both pharmacological inhibition (Pan et al., 2009) and genetic deletion (Zhong et al., 2011) of MAGL result in significant prolongation of synaptic 2-AG signaling, consistent with a role for MAGL in the termination of 2-AG-mediated CB1R signaling. In further support of this hypothesis, the MAGL inhibitor, JZL184, mimics many of the effects of CB1R direct agonists, including analgesia, hypomotility and hypothermia (Long et al., 2009). JZL184 also reduces anxiety-like behaviors (Sciolino, Zhou, & Hohmann, 2011) and protects against chronic stress-induced behavioral changes (Sumislawski, Ramikie, & Patel, 2011) in CB1R-dependent manners. Interestingly, MAGL inhibition is also associated with a significant decrease in the concentration of free arachidonic acid in brain (Nomura et al., 2011). As a result, inhibition of MAGL is associated with reduced neuroinflammation in a traumatic brain injury model (Katz et al., 2015) and may be useful for the treatment of neurodegenerative disorders, including Alzheimer's disease (Chen et al., 2012).

Early studies using prolonged or complete inhibition of MAGL activity discovered that chronic elevation of 2-AG could result in down-regulation of CB1Rs (Schlosburg et al., 2010). Clearly, this would be counterproductive as it leads to a reduction in eCB activity rather than an increase. However, low doses of irreversible inhibitors can avoid this problem (Kinsey et al., 2013); and reversible inhibitors could theoretically provide less persistent MAGL inhibition and avoid prolonged elevation of 2-AG.

MAGL inhibition will selectively enhance CB1R activation with on-going 2-AG-mediated signaling – it will not activate CB1Rs not normally targeted by endogenous 2-AG release. This differentiates MAGL inhibition from direct CB1R agonists, which can increase activity at all CB1Rs. As a result, compared to direct CB1R agonists, it is likely that MAGL inhibition could have fewer adverse or off-target effects.

With respect to the clinical development of MAGL inhibitors, Lu AG06466 is the first MAGL inhibitor in humans that has undergone Phase 1 trials for safety and target efficacy (Cisar et al., 2018). Lu AG06466 has been found to bind MAGL, inhibit 2-AG metabolism and produce elevations in 2-AG signaling in humans (Müller-Vahl et al., 2022). There has been some initial advancement into Phase 2 trials with this compound, particularly in the domain of tic disorders, although these trials have produced mixed results and beneficial effects seen in pilot work have not been replicated in Phase 2 trials (Müller-Vahl et al., 2022).

Other hydrolases can utilize 2-AG as a substrate, including *α, β* hydrolase-domain containing (ABHD)6 and ABHD12 (Blankman et al., 2007; Marrs et al., 2010, 2011). Pharmacological inhibition of ABHD6 has anti-epileptic effects in a spontaneous seizure mouse model (Naydenov et al., 2014) and improves coordination and memory performance in a traumatic brain injury model (Tchantchou & Zhang, 2013). There is no current clinical investigation of pharmacological tools targeting these enzymes and so their potential utility in humans remains unknown.

### Inactivation of AEA: hydrolysis

The primary hydrolytic enzyme acting on AEA in the brain is fatty acid amide hydrolase (FAAH) (Cravatt et al., 1996). FAAH is an intrinsic membrane protein present primarily on intracellular membranes (Hillard, Wilkison, Edgemond, & Campbell, 1995). Several studies suggest that FAAH activity is enhanced by protein kinase A-mediated phosphorylation (Gray et al., 2015; Rossi et al., 2007); however, the precise mechanism for this has not been determined.

The development of FAAH inhibitors was one of the first pharmacological interventions developed for the eCB system, with the first FAAH inhibitor, URB597, being introduced almost 20 years ago (Kathuria et al., 2003). URB597 is an irreversible inhibitor of FAAH and in the following years similar carbamate-based inhibitors were developed for preclinical testing. One such irreversible inhibitor with a carbamate structure, ASP8477, advanced into clinical trials in humans for neuropathic pain, but was not found to be effective (Bradford et al., 2017). A similar compound, ASP3652, was also found to be ineffective in a clinical trial for bladder pain (Houbiers et al., 2021). SSR411298, a reversible FAAH inhibitor with a carbamate structure, showed therapeutic potential in preclinical models of stress exposure (Griebel et al., 2018), but was not found to have any benefit in a trial for geriatric depression (Sanofi, 2013). In 2011, PF-04457845, a highly selective and specific covalent, irreversible inhibitor of FAAH was developed through activity-based protein profiling (Johnson et al., 2011). PF-04457845 underwent Phase 1 testing in humans and was found to be safe, effective at elevating AEA and did not produce any notable adverse events (including a lack of psychoactivity and euphoria, which is seen with THC) (Li et al., 2012). While this compound was found to be ineffective in treating osteoarthritic pain (Huggins, Smart, Langman, Taylor, & Young, 2012), as we will discuss later, there has been additional clinical testing of this compound which has found some potential utility in the domain of psychiatric conditions. Finally, JNJ42165279 (Postnov et al., 2018) is a urea-derived reversible FAAH inhibitor that has also undergone clinical development for affective disorders, which will be discussed in depth later in this review.

### Inactivation of eCBs: role of transport proteins

2-AG and AEA are highly hydrophobic molecules, and it is hypothesized that they require protein transporters to translocate aqueous spaces, such as cellular cytosol and the synapse (Kaczocha & Haj-Dahmane, 2021). Several binding proteins for the eCBs have been identified that could serve as chaperones or transporters. For example, several members of the fatty acid binding protein family (FABPs), including FABP5 which is present in brain, bind AEA and 2-AG (Kaczocha, Vivieca, Sun, Glaser, & Deutsch, 2012). Preclinical studies using SBFI-26, which prevents binding of eCBs to FABP5 (Hsu et al., 2017), provide evidence that FABP5 is required for some eCB activity. For example, inhibition of FABP5 reduces both tonic and phasic CB1R regulation of glutamate release in the dorsal raphe nucleus (Haj-Dahmane et al., 2018), suggesting that eCB binding to FABP5 is required for 2-AG mobilization. On the other hand, inhibition of FABP5 in a murine model of high intraocular pressure reduced pressure in a CB1R-dependent manner (Miller et al., 2020), suggesting that FABP5 is involved in eCB inactivation in this circumstance.

There is evidence that sterol carrier protein 2 (SCP-2) could also function as an intracellular transport protein for the eCBs. SCP-2 binds to both AEA and 2-AG with *K*_i_ values in the nanomolar range (Hillard et al., 2017) and overexpression of SCP-2 enhances the cellular accumulation of AEA (Liedhegner, Vogt, Sem, Cunningham, & Hillard, 2014). SCP-2 is enriched in synaptosomal preparations (Myers-Payne et al., 1996), supporting its ability to regulate eCB concentrations at synapses. Interestingly, the AEA clearance inhibitor, AM404, binds to SCP-2 (Hillard et al., 2017) which could contribute, along with FAAH inhibition (Jarrahian, Manna, Edgemond, Campbell, & Hillard, 2000), to its efficacy as an indirect CB1R agonist.

Early studies of the characteristics of neuronal accumulation of AEA supported the existence of a plasma membrane protein transporter that participates in facilitated diffusion to promote AEA movement across membranes (Hillard & Jarrahian, 2003). This concept is supported by recent studies using WOBE437 (Chicca et al., 2017). WOBE437 inhibits AEA accumulation and mimics many effects of cannabinoid agonists, including analgesic, anxiolytic and anti-inflammatory effects. Thus, WOBE437, and more importantly, its target protein, could be another avenue for the development of indirect agonists for AEA signaling. There is some recent work, as well, that pannexin channels may be able to transport AEA across lipid membranes (Bialecki et al., 2020), although this requires additional characterization.

Taken together, there are several different pharmacological options for targeting the eCB system. The direct CB1R agonist, THC, has been used by humans for thousands of years for various medicinal and recreational purposes. Although humans report that cannabis elevates mood and reduces feelings of anxiety (Andrade, Renda, & Murray, 2019; Stanciu, Brunette, Teja, & Budney, 2021), cannabis use can also result in postural instability, interference with vehicle driving skills, and abuse liability and furthermore, its use is associated with the development or exacerbation of psychiatric conditions like schizophrenia (Arnold, 2021). THC is a CB1R partial agonist – recent human experience with full efficacy agonists indicates that enhanced efficacy might produce more robust therapeutic effects but with a tradeoff of far greater risk for significant adverse effects. While there is room for improved formulations of existing direct partial agonists and for partial agonists with better oral bioavailability, it is not likely that direct agonists could be developed that are better than those already available (i.e. THC and levonantradol). CB1R antagonists, on the other hand, have gone through clinical development and trials but given the development of adverse psychiatric side effects (particularly depression and anxiety), as well as their apparent lack of efficacy in other psychiatric conditions such as schizophrenia, it seems unlikely that this class of drug will be pursued as therapeutic option for psychiatric conditions.

Indirect agonists, which inhibit inactivation of the eCBs, can avoid some of the adverse effects of a direct agonist because they enhance activation of CB1R signaling that is already engaged. This is the basis for the development of FAAH and MAGL inhibitors as eCB system-activating therapeutics. While it is still early days, the currently available data suggest that drugs with these mechanisms are safe, although their modest efficacy suggests that they may need to be combined with other therapeutics. Not surprising given the different roles of 2-AG and AEA as CB1R agonists (deRoon-Cassini, Stollenwerk, Beatka, & Hillard, 2020), preclinical studies have found that FAAH and MAGL inhibitors have non-overlapping effects [for example (Busquets-Garcia et al., 2011)] which could allow for better ‘tuning’ of the drug to the condition to be treated. The recent discoveries that both eCBs bind to transporter proteins provide additional opportunities to develop novel, indirect compounds that could potentiate or synergize with the enzyme inhibitors. PAMs also represent an appealing approach to selective activation of CB1R signaling and could have advantages over indirect agonists since they are selective for CB1R signaling, while the indirect agonists could affect all molecules that are substrates for the enzyme or transporter that is being targeted. Although promising, CB1R PAMs have not undergone Phase I testing in humans, so their adverse effect profile and clinical utility are unknown.

To date, there are three disease classes that have both strong preclinical data and some early encouraging clinical data, indicating that there may be some potential for targeting the eCB system. These diseases are substance use disorders, anxiety/stress-related psychiatric conditions and autism spectrum disorder (ASD). The remainder of this review will focus on the current state of knowledge for each of these conditions to highlight where the field is at and where it is moving.

## Substance use disorders

Preclinical studies demonstrate that modulating the eCB system influences positive reinforcement, anxiety, stress-induced craving, and relapse to substance use (Parsons & Hurd, 2015). These effects suggest that eCB-based pharmacotherapies can be developed for discrete aspects of substance use: one approach is to directly reduce the positive reinforcing and subjective effects of a substance, while another approach is to reduce withdrawal symptoms, craving and the likelihood of recurrence in people who are abstinent. With these approaches in mind, the objective of this section is to describe studies testing eCB-related medications for the treatment of the three substances with the most clinical study: cannabis, tobacco, and alcohol.

### Cannabis use disorder

Human laboratory studies have shown that the CB1R antagonist/inverse agonist, rimonabant (40, 90 mg), attenuated the subjective effects of smoked cannabis (Huestis et al., 2001, 2007). However, further study of this interaction was halted when rimonabant was shown to produce serious psychiatric side effects, such as depression, anxiety and suicidality when given repeatedly (20 mg/day) (Christensen et al., 2007).

An alternative to directly blocking cannabis's effects at the CB1R is to focus on its secondary-signaling effects. Agonists at the CB1R initiate a series of intracellular signaling cascades (Turu & Hunyady, 2010), a subset of which can be inhibited by the endogenous steroid, pregnenolone, which as described above acts as a signaling-specific inhibitor of the CB1R, and so acts independently of blocking CB1R orthosteric agonist binding (Vallée et al., 2014). Preclinical studies show that this selective inhibition decreases the positive subjective and reinforcing effects of CB1R agonists (Vallée et al., 2014).

Aelis Farma has developed a pregnenolone analog, AEF0117, that maintains the pharmacodynamic characteristics of pregnenolone but is not metabolized to active steroids, is well absorbed, has a long half-life, and does not produce behaviors associated with rimonabant (e.g. increased anxiety-like behavior, decreased food intake). We recently described evidence supporting this approach for the treatment of CUD: early-phase clinical studies with AEF0117 show that it is well tolerated in both healthy volunteers and people who use cannabis daily (no evidence of precipitating withdrawal) and reduces the positive subjective and reinforcing effects of smoked cannabis (Haney et al., 2023). These findings led support to an ongoing multi-site randomized, placebo-controlled clinical trial to test AEF0117 in patients seeking treatment for CUD.

Another approach is to reduce the likelihood of cannabis withdrawal in those who are abstinent. Daily cannabis use is associated with a range of adaptations in the eCB system, including selective and reversible downregulation of CB1Rs [e.g. (Hirvonen et al., 2012; Spindle et al., 2021)], as well as in changes in brain levels of FAAH (Boileau et al., 2016) and circulating eCBs [see (Jacobson et al., 2021) for review] relative to people who do not use cannabis. Blunted eCB activity due to downregulated CB1Rs and increased levels of FAAH may contribute to symptoms of cannabis withdrawal.

In support of this idea, orally administered CB1R agonists have been found to consistently reduce symptoms of cannabis withdrawal. These medications also improve clinical trial retention and show minimal evidence of misuse (likely due to the slow onset and long duration of oral cannabinoids), but have not been shown to significantly reduce cannabis use in clinical trials (Brezing & Levin, 2018). An important caveat to these negative clinical trials is that they mostly test patients currently using cannabis. It may be that agonist replacement will work best in those who are abstinent from cannabis to truly assess relapse prevention, but this is difficult to achieve in CUD patients.

An alternative to administering CB1R agonists is to increase concentrations of endogenous cannabinoids by blocking their enzymatic degradation. FAAH inhibition elevates AEA levels and attenuates precipitated THC withdrawal preclinically without producing evidence of THC-like intoxication (Justinova et al., 2008; Schlosburg et al., 2009). In recently abstinent men with CUD, FAAH inhibition with PF-04457845 reduced cannabis withdrawal symptoms and cannabis use (D'Souza et al., 2019). An ongoing multi-site randomized clinical trial is testing PF-04457845 as a potential treatment for CUD (D'Souza, 2022). We will soon learn whether this approach is superior to exogenous CB1R agonist approaches.

### Tobacco (Nicotine) use disorder (TUD)

Although it was not surprising that rimonabant reduced the subjective effects of cannabis, evidence that this medication also reduces the reinforcing effects of nicotine was one of many clues that targeting the eCB may have potential to treat a range of SUDs. To start preclinically, CB1R antagonists reduce nicotine reinforcement [see (Saravia, Ten-Blanco, Pereda-Pérez, & Berrendero, 2021; Spanagel, 2020)], presumably by blocking eCBs from disinhibiting dopaminergic neurons in the ventral tegmental area [see (Asth, Santos, & Moreira, 2022; Butler & Le Foll, 2020)]. These findings appear consistent with human imaging data [summarized by (Hirvonen et al., 2018)] – with cannabis, THC acts on CB1Rs directly and thereby downregulates CB1Rs in neocortical brain regions but not in the basal ganglia, midbrain, or cerebellum. People who use tobacco, by contrast, have downregulated CB1Rs in all brain regions assessed, perhaps reflecting widespread, repeated stimulation of endocannabinoid signaling by nicotine.

In a large clinical trial (*n* = 2097), rimonabant administration (20 mg/day, but not 5 mg/day, for 10 weeks) produced significantly greater tobacco abstinence rates and less weight gain relative to placebo. Yet as was seen in the metabolic disorder studies, rimonabant also increased the incidence of adverse events (e.g. nausea, diarrhea, anxiety), and the likelihood of suicidality compared to placebo when taken for 1 year (Robinson et al., 2018). Although these findings eliminate rimonabant as an option, the data nonetheless suggest that further investigation of eCB medications is warranted as a potentially novel approach to smoking cessation treatment.

Perhaps counter-intuitively, FAAH inhibitors, which increase CB1R agonist activity rather than block it like rimonabant, also attenuated both nicotine self-administration and reinstatement of nicotine seeking in rats and non-human primates, and decreased nicotine-induced dopaminergic activity [see (Sagheddu, Torres, Marcourakis, & Pistis, 2020)]. Alternately, aside from metabolizing AEA, FAAH also metabolizes a family of fatty acid ethanolamides which do not act on CB1Rs but could still be relevant in the potential actions of FAAH inhibitors in TUD. Preclinical studies show that palmitoylethanolamide (PEA) and oleoylethanolamide (OEA), both of which are elevated following inhibition of FAAH, reduce nicotine reward via their effects on peroxisome proliferator-activated receptor (PPAR)*α*, likely by reducing nicotine's effects on dopamine neurons [see (Melis & Pistis, 2014; Sagheddu et al., 2020)]. Although the PPAR*α* agonists, gemfibrozil and fenofibrate, did not reduce tobacco use clinically (Gendy et al., 2018; Perkins et al., 2016), these medications have poor brain penetrability, so this approach cannot be ruled out as a potential novel treatment for TUD (Sagheddu et al., 2020). Thus, there appears to be significant potential for medications that either target the eCB or target the same family as eCBs to treat TUD. To our knowledge, FAAH inhibitors have not yet been tested clinically for the treatment of TUD.

### Alcohol use disorder (AUD)

Individuals with AUD have widespread downregulation of the CB1R in both cortical and subcortical brain regions that do not appear to reverse even after 4 weeks of abstinence (Hirvonen et al., 2013). As noted above for tobacco cigarette smokers, the pattern of CB1R downregulation supports the hypothesis that chronic alcohol use downregulates CB1Rs by increasing endogenous cannabinoid activity (Hirvonen et al., 2018). In addition to CB1R downregulation, patients with AUD in early abstinence (3–7 days) have reduced brain FAAH levels and elevated levels of AEA and OEA in plasma relative to healthy controls, which appeared to recover after 2–4 weeks of abstinence (Best et al., 2020). These data suggest that alcohol alters eCB activity and that the eCB system is a potential target for the treatment of AUD.

In support, preclinical studies show that CB1R antagonists, including those restricted to the periphery, reduce alcohol self-administration [for review, see (Godlewski et al., 2019; Sagheddu et al., 2020; Soyka et al., 2008)]. However, this effect has not been translated clinically. Rimonabant, at the same dose shown to reduce tobacco cigarette use (20 mg/day), was tested in a double-blind, placebo-controlled study in recently detoxified alcohol-dependent patients. In the rimonabant group, 41.5% relapsed compared to 47.7% in the placebo group. Consistent with rimonabant's effects in other populations, the AUD patients taking rimonabant lost more weight than those receiving placebo (Soyka et al., 2008). The number of adverse events, however, did not vary as a function of dose in this study.

As with TUD, there is preclinical evidence that the fatty acid ethanolamide, OEA, reduces measures of alcohol withdrawal and alcohol-seeking [see (Orio, Alen, Pavón, Serrano, & García-Bueno, 2018; Sagheddu et al., 2020)]. Preclinical studies also show that PPAR*α* agonists, alone or in combination with CB1R antagonists, reduce alcohol self-administration or voluntary drinking [see (Sagheddu et al., 2020)]. There are no published clinical studies with PPAR*α* agonists to date but they appear to be warranted (Matheson & Le Foll, 2020).

To conclude, the eCB system is clearly involved in the mechanism by which a range of substances produce their reinforcing effects. CB1R antagonists directly attenuate the positive subjective effects of cannabis, but also reduce the rewarding and reinforcing effects of non-cannabinoid substances, likely by blocking eCBs from disinhibiting dopaminergic neurons in the ventral tegmental area (Asth et al., 2022; Soyka et al., 2008). Studies by Mantsch and colleagues suggest that another potential use for CB1R antagonists could be in the prevention of stress-induced relapse to drug taking (McReynolds et al., 2018, 2016). The mechanism involves glucocorticoid mobilization of eCB signaling, which disinhibits PFC to ventral striatal afferents and potentiates reward-driven behavior. The significant adverse effects produced by orthosteric CB1R antagonists, however, suggest that blocking the CB1R is not a feasible long-term pharmacotherapy because tonic eCB activity is essential for mood and reward processing. Their potential usefulness as a short-term therapy has not been explored.

There is some preclinical evidence to suggest that a component of rimonabant's mood-related adverse effects may reflect its inverse agonist properties, as neutral CB1R antagonists decrease nicotine, alcohol and heroin self-administration without producing the same anxiety-like behavior in animals [see (Galaj & Xi, 2019)]. However, as mentioned above, there are also preclinical data indicating that tonic eCB-mediated signaling is essential for the maintenance of mood, particularly hedonia (Bluett et al., 2017), so this strategy may not extend to non-anxiety adverse effects. NAMs and signaling-specific inhibitors of the CB1R appear to be well-tolerated and reduce cannabis intoxication and use without producing the adverse effects associated with rimonabant. Reducing cannabis intoxication may be a particularly fruitful approach for the treatment of CUD, as the majority of patients seeking treatment continue to use cannabis, so directly reducing its effects may facilitate a reduction in use. FAAH inhibitors are also of considerable interest for CUD treatment, perhaps most efficaciously for those who are abstinent from cannabis use to reduce the likelihood of recurrence. An ongoing multi-site trial will be highly informative regarding this approach.

Overall, given the compelling preclinical data on the potential for medications that target the eCB system or related molecules to treat SUDs, more medications available for clinical research are needed to translate these findings into clinical studies and potentially develop novel approaches for SUD treatment.

## Stress-related psychiatric disorders

The eCB system is well established to be a central regulator of stress responsivity and can influence activity through distributed neural circuits important for emotion and affect (Morena, Patel, Bains, & Hill, 2016). Preclinical work has detailed a complex, but largely consistent, role for eCB in both the basal regulation of stress and affect in the absence of any threats, as well as an important buffer system limiting the magnitude and duration of stress responses once an aversive stimuli or experience is encountered (Morena et al., 2016). Within the amygdala, there is constitutive signaling of AEA at CB1Rs on glutamatergic terminals which constrains excitatory transmission in both the basolateral and central nuclei of the amygdala (Bedse et al., 2017; Natividad et al., 2017; Yasmin et al., 2020). In response to stress exposure, the rapid release of corticotropin-releasing hormone causes a rapid induction of FAAH activity, which in turn results in a depletion of the synaptic signaling pool of AEA (Gray et al., 2015; Natividad et al., 2017). This loss of AEA signaling at excitatory synapses results in an increase in glutamate release (Yasmin et al., 2020), which then increases the activity of post-synaptic output neurons. This loss of AEA signaling within the amygdala is sufficient to trigger the generation of a behavioral state of anxiety and may also contribute to the activation of a stress response (Morena et al., 2016). Accordingly, genetic or pharmacological inhibition of FAAH can prevent this rapid loss of AEA signaling from stress (Hill et al., 2013; Mayo et al., 2020a), and in turn, can counter many effects of different types of stress, including elevations in anxiety (Bedse et al., 2017; Bluett et al., 2014; Campos, Ferreira, Guimarães, & Lemos, 2010; Danandeh et al., 2018; Griebel et al., 2018; Haller, Goldberg, Pelczer, Aliczki, & Panlilio, 2013; Hill et al., 2013), inhibition of fear extinction (Ganon-Elazar & Akirav, 2009; Morena et al., 2018), suppression of feeding (Sticht et al., 2019) and activation of the hypothalamic-pituitary-adrenal (HPA) axis (Bedse et al., 2014; Hill et al., 2009; Navarria et al., 2014; Patel, Roelke, Rademacher, Cullinan, & Hillard, 2004).

In addition to these anti-stress actions of FAAH inhibitors, AEA signaling also plays an important role in fear regulation. Fear extinction training has been found to elevate AEA signaling in the amygdala (Gunduz-Cinar et al., 2013; Marsicano et al., 2002; Morena et al., 2021), and blockade of the CB1R can impair normative fear extinction while FAAH inhibition can augment fear extinction (Chhatwal et al., 2009; Chhatwal, Davis, Maguschak, & Ressler, 2005; Fidelman, Mizrachi Zer-Aviv, Lange, Hillard, & Akirav, 2018; Gunduz-Cinar et al., 2013; Marsicano et al., 2002). Taken together, the current body of preclinical work indicates that AEA signaling can counter stress, fear and anxiety, largely through its ability to gate excitability of neurons in the amygdala.

Translational studies have found very similar effects in humans who have elevations in AEA signaling. The most abundant source of evidence in humans comes from genetic studies. The FAAH gene in humans is known to possess a relatively common, functional single nucleotide polymorphism (SNP) in which a cytosine is replaced by an adenine, resulting in an amino acid substitution in the protein where a proline is replaced by a threonine (typically referred to as the C385A or P129T SNP) (Chiang, Gerber, Sipe, & Cravatt, 2004). The variant of FAAH seen in the C385A SNP exhibits increased proteolytic degradation relative to the native form of the protein, resulting in destabilization of the protein and a consequential 40–50% reduction in protein expression of FAAH (Chiang et al., 2004; Dincheva et al., 2015). This reduction in FAAH expression results in a moderate elevation of AEA signaling (approximately a 20% elevation in AEA levels) (Chiang et al., 2004; Dincheva et al., 2015). As such, humans who possess the rare variant of the C385A FAAH SNP represent a population who have elevations in AEA signaling. Consistent with what has been found in the rodent literature, humans bearing the C385A FAAH SNP exhibit lower levels of trait anxiety, blunted reactivity of the amygdala in response to threat, increased functional and structural connectivity between the ventromedial prefrontal cortex and the amygdala and enhanced fear extinction (Demers, Drabant Conley, Bogdan, & Hariri, 2016; Dincheva et al., 2015; Gärtner et al., 2019; Gee et al., 2016; Green et al., 2021; Gunduz-Cinar et al., 2013; Hariri et al., 2009; Sisk et al., 2022; Zabik et al., 2022). Individuals with PTSD who possess this FAAH SNP also exhibit reduced symptoms of arousal and blunted stress-induced anxiety (Spagnolo et al., 2016), as well as enhanced treatment responses to virtual reality-assisted psychotherapy with D-cycloserine augmentation, particularly in those who have comorbid major depression and PTSD (Difede et al., 2022). As such, this represents a rare case of positive translation from basic science rodent studies to human subjects with genetic variance, where all the converging evidence indicates that elevations in AEA signaling confers some resilience to the effects of stress, particularly with respect to anxiety, and that these effects likely relate to blunted neural activity in the amygdala (Mayo, Rabinak, Hill, & Heilig, 2022).

The development of FAAH inhibitors in humans has led to further clinical investigation regarding the potential of FAAH inhibitors. Four days of administration of the reversible FAAH inhibitor JNJ-42165279 was found to blunt activation of the amygdala in response to threat cues in healthy humans (Paulus et al., 2021), paralleling what was found in humans who possess the FAAH C385A SNP (Hariri et al., 2009) and in animal studies showing that FAAH inhibitors can blunt stress-induced increases in excitatory neurotransmission within the amygdala (Yasmin et al., 2020). This outcome is consistent with other classic anxiolytic drugs. This study, however, did not find any significant impact of FAAH inhibition on fear conditioning or extinction. A second similar study performed in healthy research participants utilized 10-day treatment with the irreversible FAAH inhibitor PF-04457845. At the conclusion of the 10-day administration, participants were exposed to a stress challenge and fear conditioning and extinction. FAAH inhibition was found to dramatically reduce stress-induced autonomic responses and moderately dampen stress-induced affective changes while having no impact on the HPA axis response to stress (Mayo et al., 2020b). More so, unlike the previous study, here FAAH inhibition was found to augment fear extinction while having no impact on fear conditioning itself (Mayo et al., 2020b). As such, these short-term pharmacological studies in humans generally support the previous literature and suggest that there may be some therapeutic potential for FAAH inhibition for the management of anxiety and stress-related psychiatric disorders.

In line with this prediction, there have been some initial clinical trials in disease states with the FAAH inhibitors which encourage further work. First, as previously mentioned in the SUD section, FAAH inhibition has been examined in the context of CUD and while the primary outcomes of that study related to the recurrence of cannabis use, it is important to note that elevations in anxiety seen following the cessation of cannabis were found to be significantly attenuated by treatment with the PF-04457845 FAAH inhibitor (D'Souza et al., 2019). A 12-week multi-center trial for social anxiety disorder was also performed with the JNJ-42165279 FAAH inhibitor. Within this trial, there was no significant main effect of the FAAH inhibitor in social anxiety disorder; however, the proportion of research participants who experienced a much improved or very much improved end point score on their Clinical Global Inventory was significantly increased for those who had been treated with the FAAH inhibitor (Schmidt et al., 2021). While this outcome was minor, it is important to note that some of this lack of effect may be related to pharmacokinetics of the drug. Specifically, it was found that for the majority of participants on the FAAH inhibitor, elevations in plasma AEA were not seen, suggesting sub-optimal dosing. Interestingly however, once participants were stratified based on whether their trough levels of AEA were increased, it was found that those who exhibited the largest increases in AEA following treatment with the FAAH inhibitor also exhibited the greatest reduction in anxiety symptoms (Schmidt et al., 2021). As such, while this trial suffered from technical issues, the outcome data do suggest some potential benefit of FAAH inhibition in the context of treating social anxiety disorder. There is currently an ongoing trial with this FAAH inhibitor for PTSD (clinical trial EudraCT 2020-001965-36), as well as a large multi-site trial for PTSD with PF-04457845 (now called JZP150 since acquisition of this compound by Jazz Pharmaceuticals; clinical trial NCT05178316). The outcomes of both of these trials will be very informative with respect to the potential of FAAH inhibition for the treatment of PTSD.

While there is some optimism for a potential role for FAAH inhibition in the treatment of PTSD and anxiety disorders, the same cannot be said for depression, another stress-related psychiatric disorder. As mentioned earlier, a trial from Sanofi looking at FAAH inhibition for the treatment of geriatric depression was not found to produce benefit. More so, in tandem with the social anxiety disorder trial discussed above, a trial for JNJ-42165279 for anxious depression was also run, but similarly was found to produce no benefit (Janssen Research & Development, LLC, 2022). As such, it seems unlikely that there will be a therapeutic role for FAAH inhibitors in the treatment of depression.

## Autism spectrum disorder

ASD is one of the newest areas to explore the utility of eCB modulation for therapeutic intervention. ASD is a neurodevelopmental disorder that is behaviorally diagnosed through the observation of impairments in social functioning and the presence of restricted, repetitive behaviors (a category which includes atypical sensory reactivity). Despite the well-defined behavioral diagnostic features of ASD, the biological markers, etiology, and contributing pathophysiological mechanisms of this brain disorder remain unclear. Importantly, there is a high prevalence of co-occurring conditions such as, intellectual disability, anxiety, seizure susceptibility, developmental delays, sleep disturbances, gastrointestinal disorders, and attention-deficit/hyperactivity disorder with ASD which results in a highly heterogenous clinical population and a complicated landscape for the development of pharmacological interventions (Neumeyer et al., 2019). Since the eCB system it is directly and indirectly involved in the range of behaviors that are also core and co-occurring ASD features, it is an attractive inroad for the development of interventions that are well-fitted to the complexity of the ASD clinical population. Data from preclinical studies in models of ASD provides robust support for targeting the eCB system, particularly in ameliorating specific features such as social impairments. Given that the etiopathogenesis of ASD involves a combination of genetic, environmental, and idiopathic factors, data from studies with these etiologies will be overviewed to highlight specific targets of engagement.

Fragile X syndrome (FXS) accounts for approximately 1 to 6% of all ASD cases making it the most common, known monogenetic form of ASD (Kaufmann et al., 2017). It is considered a classic example of syndromic ASD, which are cases of ASD in individuals with pre-existing neurological disorders. In the human clinical population, individuals with a dual diagnosis of ASD and FXS are more affected in cognitive and behavioral domains compared to those with ASD or FXS alone (Kaufmann et al., 2017). Importantly, in addition to the presence of autistic features (e.g. tactile defensiveness, hand flapping, and social communication deficits), a hallmark of FXS is the presence of intellectual disabilities and developmental delays (IDDs). Since IDDs significantly impact the long-term outcomes and quality of life for autistic individuals, identifying the specific eCB biology in this model that contributes to the co-occurrence of ASD and IDD is highly relevant to improving outcomes for an underserved stratum of the ASD population. The Fmr1 knockout (KO) mouse model, which lacks the Fragile X (FMRP) protein, results in a feature profile that closely resembles the behavioral and physiological clinical presentation of FXS patients (i.e. cognitive deficits, repetitive behaviors, altered social behaviors, structural changes in dendritic spines, and atypical neurotransmission). In Fmr1 KOs, the four most studied components of the eCB system (i.e. AEA, 2-AG, CB1R, and CB2R) have been systematically evaluated for their potential as targets of engagement for pharmacological intervention. URB597, a FAAH inhibitor, has been used in two different mouse backgrounds (i.e. C57BL/6J and FVB/NJ) to modulate AEA signaling (Qin et al., 2015; Wei et al., 2016). In the C57 background, AEA improved cognitive impairments and anxiety phenotypes in Fmr1 KOs (Qin et al., 2015). On the FVB background, modulation of AEA signaling produces a full reversal of social behavior impairments in Fmr1 KOs (Wei et al., 2016). The mechanism underlying these disparate effects between these studies is unclear, but the results suggest that genetic background and timing of FAAH inhibition are important factors in mediating the specificity of effects on behaviors from modulating AEA signaling. In contrast to the number of studies assessing AEA modulation in Fmr1 KOs, only one study has assessed 2-AG signaling. Blockade of 2-AG degradation through MAGL inhibition with JZL184 decreased anxiety (i.e. decreased activity in an open field and increase aversion of open arm in elevated plus maze) and normalized locomotor activity (Busquets-Garcia et al., 2013; Jung et al., 2012). Similar effects on anxiety have been observed through modulation of CB2R activity with AM630 (Busquets-Garcia et al., 2013). Finally, changes in CB1R activity have been consistently linked to cognitive function in the Fmr1 KO model of ASD. While both chronic and acute administration of rimonabant, the CB1R antagonist, improved cognitive impairment, only acute administration led to improved pain desensitization in Fmr1 KOs. In summary, in Fmr1 KOs, modulation of AEA signaling largely mitigates impairments in social behaviors, altering 2-AG signaling and CB2R activity impacts anxiety, and modulating CB1R activity improves cognition. While more work is needed to clarify whether the preclinical findings of generalized changes in AEA signaling and localized changes in CB1R activity in distinct neuroanatomy can be achieved simultaneously in humans, the data offer a potential inroad for treating a severely underserved stratum of the ASD clinical population.

Nonsyndromic forms of ASD are cases that are not linked to any pre-existing neurological disorder and can stem from rare *de novo* mutations in candidate genes like neuroligin (NLGN). NLGNs are postsynaptic cell adhesion molecules that regulate synaptic transmission and neuronal development (Nguyen, Lehr, & Roche, 2020; Rothwell et al., 2014). While humans have five isoforms of NLGNs, only mutations in NLGN3 and NLGN4 have been found in ASD cases (Jamain et al., 2003; Nguyen et al., 2020). Two rodent models of NLGN3 mutations [i.e. the NLGN3 KO and NLGN3R451C knock-in (KI)] (Rothwell et al., 2014; Tabuchi et al., 2007). These NLGN models also have disrupted tonic eCB signaling in hippocampal cholecystokinin-expressing basket cells as well as increased distribution of CB1Rs in the hippocampus and cortex (Földy, Malenka, & Südhof, 2013; Speed, Masiulis, Gibson, & Powell, 2015; Zamberletti, Gabaglio, & Parolaro, 2017). Behaviorally, both loss- and gain-of-function NLGN3 mutations alter social behaviors, but the constellation of social behaviors affected varies with the NLGN mutation. NGLN3 KOs demonstrate impaired social communication (i.e. impaired ultrasonic vocalizations), altered social behaviors (i.e. deficits in social novelty preference), and atypical sensory reactivity (i.e. reduced olfactory function), the canonical ASD behavioral triumvirate (Jamain et al., 2003; Rothwell et al., 2014). Conversely, NLGN3 KIs have impaired social interactions, enhanced spatial learning, exhibit repetitive, restricted behaviors (i.e. stereotypical object exploration), and heightened aggression (Burrows et al., 2015). While aggression is not a core ASD feature, it is present in 39.5% of autistic individuals and is one of two druggable targets in ASD with an FDA-approved pharmacological therapy (Cossio, Stadler, Michas, Johnston, & Lopez, 2020; Hosie et al., 2018). Use of CB1R agonist, WIN 55, 212-2, reduced aggressive behaviors (i.e. attack incidences, duration, and increased attack latency) in NLGN3 KIs replicating previous data showing CB1R KO mice are more aggressive compared to wildtype littermates and that CB1R agonists modulate aggressive behaviors (Burrows et al., 2015; Hosie et al., 2018; Rodriguez-Arias et al., 2013; Rodríguez-Arias et al., 2015). Findings from the NLGN3 models offer several new insights relevant to drug development for nonsyndromic ASD cases. First, the NLGN3 models further corroborate a role of AEA signaling in ASD-related social behaviors and the influence of genetic background on the constellation of social behaviors affected. These models also expand on the neural circuitry and synaptic architecture that when perturbed by eCB dysregulation produce distinct social behavioral profiles. While it remains unclear how these findings can be combined with those from syndromic ASD models with eCB regulation, they do inform future eCB-based precision medicine efforts. Second, two different studies in NLGN3 KIs found that modulating CB1R activity in the amygdala reduced aggressive behaviors (Burrows et al., 2015; Hosie et al., 2018). Since currently prescribed pharmacotherapeutics for aggression increase the incidence of distressing off-target effects (i.e. weight gain, gynecomastia, and hyperphagia) novel pharmacotherapies that target amydalar CB1R activity may offer a less fraught intervention option. Finally, repetitive, restricted behaviors are one of the most intractable and complex features of ASD. So, the link between NLGN and repetitive, restricted behaviors highlights a highly relevant target of engagement for pharmacological intervention development.

Apart from syndromic and nonsyndromic cases of ASD, recent evidence has suggested that between 40 and 50% of variance in ASD cases is related to environmental factors. Prenatal exposure to valproic acid (VPA) is the most well-studied environmental factor model of ASD and has been extensively used to investigate eCB dysregulation in ASD. Behaviorally, VPA-exposed animals have deficits in social communication, social play, obvious stereotypies, and increased anxiety along with changes in AEA and 2-AG tone. In one VPA model, changes in DAGL and MAGL were found alongside behavioral changes in sociability, anxiety, and nociception (Kerr, Downey, Conboy, Finn, & Roche, 2013). Social exposure in these animals led to increased hippocampal levels of AEA and other fatty acid ethanolamides, OEA and PEA (Kerr et al., 2013). Other studies have shown decreased NAPE-PLD and increased FAAH significantly impacting levels of AEA across the whole brain (Servadio et al., 2016). These changes in AEA metabolism were present across the lifespan (i.e. from infancy to adulthood) and are accompanied by changes in CB1R activity in the amygdala, hippocampus, and dorsal striatum – all areas that contribute to socio-emotional processing (Servadio et al., 2016). While pharmacological intervention with URB597 or PF-3845 to modulate AEA reversed social impairments, FAAH inhibition also increases PEA and OEA, enhancing the activity of hippocampal CB1Rs and effecting cognition (Babayeva, Assefa, Basu, & Loewy, 2022; Kerr et al., 2013; Kerr, Gilmartin, & Roche, 2016; Servadio et al., 2016; Trezza et al., 2012; Trezza & Vanderschuren, 2008). In sum, the VPA models replicate data in other ASD animal models linking AEA signaling and social functioning and expand what is known by providing important considerations regarding the impact of development on AEA modulation. For neurodevelopmental disorders like ASD, understanding the precise timing for eCB modulation is critical to the development of pharmacotherapies if provided during a specific window could eliminate the need for drug intervention across the lifespan.

The majority of ASD diagnoses stem from idiopathic etiologies, which are typically modeled with inbred BTBR T + tf/J mice. These animals show a variety of behaviors that are reminiscent of the core ASD features such as impairments in social functioning (i.e. deficits in social approach, social transmission of food preference, social interactions, and social play), diminished ultrasonic vocalizations, and increased self-grooming (Meyza et al., 2013; Onaivi et al., 2011). The behavior changes are seen in addition to increased CB1R density and upregulation of CB1R function in the hippocampus and cortex compared to control mice (Gould et al., 2014, 2012). Although the social profile of BTBR mice aligns with core characteristics in ASD, only two studies have assessed eCB manipulation in this model. In one study, the AEA reuptake inhibitor, AM404 (i.e. acetaminophen), improved sociability (i.e. social approach and social preference) in BTBR mice by increasing AEA in the frontal cortex (Gould et al., 2012). Interestingly, the use of WIN 55,212-2 (a CB1R agonist) in BTBR mice did not increase social but it did reduce marble burying, which is often used as a proxy for repetitive, restricted behaviors in ASD (Gould et al., 2012). A more recent study found that the blockade of FAAH substantially increased levels of AEA and completely reversed social impairment (Wei et al., 2016). Blockade of CB1R prevented the improvement of social behaviors by FAAH inhibition (with URB597) indicating that AEA accumulation was acting through CB1Rs to mediate the restoration of typical social behavior (Wei et al., 2016). Findings in the BTBR model corroborate other evidence associating AEA signaling with social behavior and CB1R activity with restricted, repetitive behaviors. Given the unique profile of eCB changes identified by BTBR mice compared to other in socially impaired mice strains, these data also suggest that treatment of idiopathic ASD case may be based upon eCB system changes (Gould et al., 2012).

Collectively, these varying animal models of ASD have some common outcomes. FAAH inhibition with URB597 or AM404 are effective pathways to modulate AEA signaling and producing changes in social domain across etiopathologies of ASD. However, the given the breadth of social functions altered by AEA signaling, more work is needed to understand how to achieve specific and sensitive modulation of social behaviors. Inhibition of 2-AG signaling with JZL184 and CB2R activity with AM630 modulates anxiety and motor ability. Finally, CB1R modulation largely results in amelioration of cognitive deficits although alterations in CB1R activity can also impact aggression and social behaviors.

Compared to the range of data available on the pharmacological approaches to target the eCB system in ASD animal models, there are considerably fewer studies that have translated these preclinical findings on eCB biology to the autistic human population. The first reports of eCB dysregulation in autistic humans solely assessed cannabinoid receptor and enzyme expression. Siniscalco and colleagues, showed that in peripheral blood mononuclear cells collected from autistic children mRNA and protein levels of CB2Rs were upregulated compared to neurotypical controls (Siniscalco et al., 2013). The autistic pediatric cohort also demonstrated slightly decreased mRNA levels of NAPE-PLD (Siniscalco et al., 2013). Authors suggested the results support theories of inflammation and neuroimmune dysregulation in ASD (Brigida, Schultz, Cascone, Antonucci, & Siniscalco, 2017). Following studies evaluating the eCB system in ASD have focused on the measurement of the principal eCBs (i.e. AEA and 2-AG) and other prominent fatty acid ethanolamides (i.e. OEA and PEA). Karhson and colleagues assessed levels of AEA in peripheral blood samples collected from a prepubescent pediatric cohort (aged 3 to 12 years). Compared to controls, autistic participants had lower levels of plasma AEA, and the likelihood of an ASD diagnosis was associated with a fourfold decrease in risk with each twofold increase in plasma AEA levels (Karhson et al., 2018). These data were replicated and extended by Aran and colleagues, who assessed AEA, 2-AG, OEA, and PEA, in the serum collected from a cohort of children aged 5.5 to 21 years old. Serum levels of AEA, OEA, and PEA, but not 2-AG, were lower in autistic participants compared to typically developed children. Levels of AEA were correlated with age and body mass index such that younger autistic children with lower body weights had lower levels of serum AEA (Aran et al., 2019). Notably, although this study evaluated the relationship between eCB levels and scores on behavioral assessments, no significant relationships were found, which differs from preclinical data. The most recent report of eCB dysregulation in autistic humans is a study by Zuo and colleagues. In this case-control study, researchers assessed eCBs and fatty acid ethanolamides (i.e. 2-AG, AEA, PEA and OEA), cannabinoid receptors (i.e. CB1Rs and CB2Rs) and related enzymes (i.e. NAPE-PLD, FAAH, DAGL and MAGL) in autistic children aged three to twelve years. In addition to replicating previous results, the authors also examined the relationship between 2-AG signaling and autistic behaviors. Autistic children had lower plasma levels of 2-AG, AEA, PEA, and OEA. Peripheral blood mononuclear cells from autistic children also demonstrated significantly higher mRNA and protein levels for CB2R, FAAH, and MAGL compared to neurotypical controls (Zou et al., 2021). Moreover, PEA, which is structurally similar to AEA and synthesized by the same enzyme (i.e. NAPE-PLD), was negatively correlated with the total scores of the Autism Behavior Checklist (ABC), such that children with lower levels of PEA exhibited more severe ASD symptoms. This study is the first to demonstrate a relationship between human autistic features and paracannabinoid levels. However, given that the relationship was between total scores on the ABC, which is not a gold-standard diagnostic instrument, and PEA, the specific relationship between ASD diagnostic symptoms and PEA is unclear. Preclinical studies in ASD models which have observed changes in PEA suggest this change may be associated with atypical social behaviors (Bertolino et al., 2017; Cristiano et al., 2018; Kerr et al., 2013). In summary, while there are only a few human studies that have investigated the role of the eCB system in ASD pathophysiology, replicating of preclinical findings in humans data suggests that precision medicine efforts in ASD would be well served by supporting further research into the behavioral impact of eCB manipulation. To date, manipulations of eCB system in ASD in humans have focused on the use of phytocannabinoids in ASD. All the US-based clinical trials and international observational studies of pharmacological manipulation of the eCB system in ASD evaluated the safety, tolerance, and efficacy of compounds that include cannabidiol (CBD). The mechanism of action of CBD falls outside the scope of this review as it does not act on the canonical components of the eCB system mentioned previously. Thus, this review will not include an overview of the findings from studies that have assessed the impact of phytocannabinoids in the ASD human clinical population.

The studies reviewed indicate major progress of cannabinoid treatment in ASD; however, it is still unclear which cannabinoids are well suited to target core features of ASD. Moreover, since only exogenous cannabinoids have been tested in this clinical population the effectiveness of direct modulation of eCB signaling is unknown. Studies in co-occurring conditions, like social anxiety which directly modulate AEA signaling through FAAH inhibition, may offer greater specificity of target engagement without activating the euphorigenic or off-target effects commiserate with exogenous cannabinoid intervention (Ahmed et al., 2020; Schmidt et al., 2021). The clinical examination of FAAH inhibitors in ASD is needed, and is anticipated to occur in the near future, to directly evaluate the potential for this treatment approach in autistic humans. Additionally, there are no studies that have explored how targeting eCB dysregulation can ameliorate non-social core features of ASD (i.e. repetitive, stereotyped behaviors or atypical sensory reactivity). Thus, more research is needed to fully explore the complexity of eCB dysregulation in ASD to identify which cannabinoids are best suited to treat the range of behaviors in ASD in a symptom-specific manner and uncover the mechanisms that underlie core features.

## Conclusions and future directions

**Figure 1.**
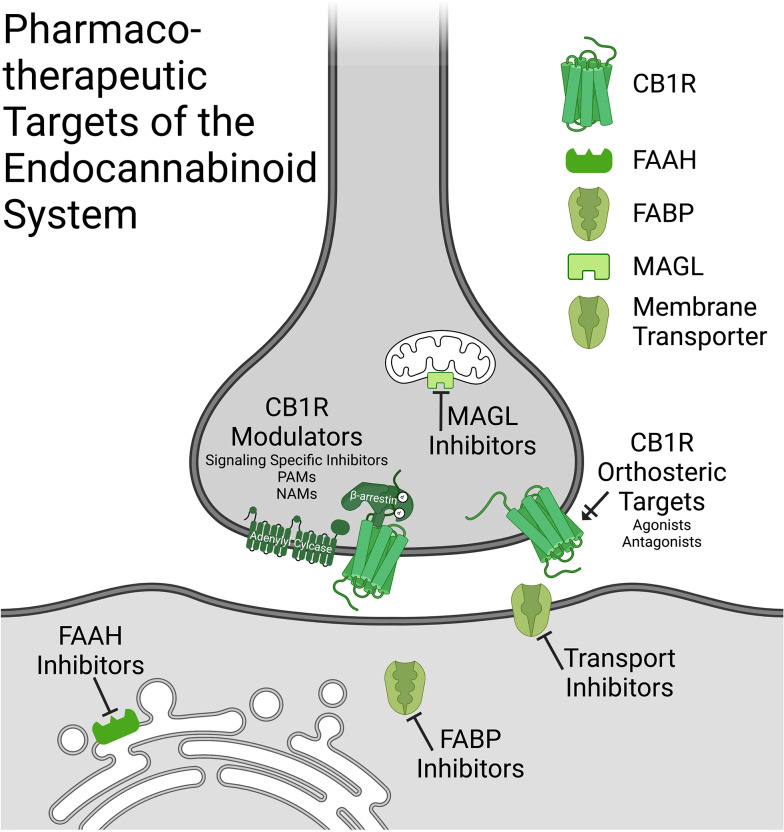
Pharmacotherapeutic targets of the endocannabinoid system. A schematic showing potential therapeutic targets of the endocannabinoid system in the central nervous system displayed in their pre- and postsynapse localization in neurons (this is not to say that central mechanisms of action could also occurs through nonneuronal cell types). Compounds include: CB1R orthosteric targets (including agonists and antognists), CB1R modulators (signaling specific inhibitors, PAMs and NAMs), FAAH inhibitors, MAGL inhibitors, FABP inhibitors and membrane transporter inhibitors. CB1R-cannabinoid receptor type 1; PAM-positive allosteric modulator; NAMnegative allosteric modulator; FAAH-fatty acid amide hydrolase; MAGL-monoacylglycerol lipase; FABP-fatty acid binding protein. Created in Biorender.

The eCB system represents a novel target for the development of a new class of drugs for psychiatric illnesses that has not previously been explored in depth. While still in the early days of translation to humans and clinical trials, preclinical data have been very encouraging and the initial studies in humans have largely supported the predictions made from rodent work. As discussed in this paper, there are several key areas where this development has advanced significantly, with some promise. More so, there appear to be several targetable components of the eCB system that have provided some optimism (see Fig. 1). First, to date it appears that FAAH inhibitors as a class of drug likely represent the most promise for translation to a novel therapeutic tool. Despite initial failures with pain, promising studies have emerged in humans with respect to CUD, social anxiety disorder and stress-related conditions such as PTSD. More so, while not tested in a clinical setting as of yet, the preclinical work in ASD, coupled to the somewhat positive findings seen using direct cannabinoid agonists or CBD, which may also potentiate AEA signaling (Leweke et al., 2012), indicate that future clinical work determining if FAAH inhibitors have benefit for ASD is urgently needed. One of the primary advantages of FAAH inhibitors is that they lack psychoactivity, unlike cannabinoids such as THC, suggesting that they would not possess the substance use disorder potential seen with some other cannabinoids. More so, the lack of any psychoactive side effects may also make this pharmacological approach more manageable as it would not have the same impact on workplace safety and driving that is seen with cannabis or intoxicating cannabinoids. On the flip side, however, FAAH inhibitors do not produce robust effects like the psychoactive cannabinoids do and that may limit their efficacy to some degree, as has been hypothesized with respect to their lack of benefit in pain conditions. One approach to potentially counter this could be the addition of a CB1R PAM, which could amplify the cellular actions of AEA at CB1Rs. To date, no PAMs have gone into testing in humans, so this remains speculative, but if FAAH inhibitors do move forward in clinical development this approach may be considered down the line.

MAGL inhibitors are still in very early stages of clinical development, so it will be important to see how data from future trials comes out as while 2-AG is a more efficacious agonist at CB1R then AEA is, that may in turn bring issues related to psychoactivity and CB1R desensitization that have not been present with FAAH inhibitors. Currently, most of the focus of MAGL inhibitors has been on neurological and neuroinflammatory conditions, however this therapeutic application could still hold benefit for psychiatric conditions, many of which are associated with neuroinflammation, such as major depression.

While CB1R antagonists are too problematic as straight therapeutic approach, as was learned from the rimonabant trials and the subsequent development of adverse psychiatric side effects, more tailored approaches to dampening CB1R signaling could hold some promise. A recently developed analog of pregnenolone has shown some positive outcomes for reducing cannabis intoxication and could be a novel approach to treating CUD as well. A multi-site clinical trial is currently underway to evaluate this specifically. Interestingly, there is also clinical development with these agents for the management of cognitive issues related to neurodevelopmental disorders and so these agents may also represent a novel drug class for disorders including ASD.

While there may still be a place for direct CB1R agonists for the treatment of conditions such as chronic neuropathic pain and multiple sclerosis, the potential risk these may carry for exacerbating psychiatric conditions, particularly psychosis and schizophrenia and possibly bipolar disorder, will likely limit their utility for the treatment of psychiatric conditions.

Overall, this is an exciting time for eCB-based therapeutics, with several recent positive trials emerging for psychiatric conditions with drugs that amplify eCB activity, there is a renewed interest in the therapeutic potential of this system. While disorders such as major depression, bipolar disorder and schizophrenia may not represent psychiatric illnesses that will benefit from this approach, there is some optimism now that SUD, anxiety and other non-major depression stress-related psychiatric disorders and ASD may represent a cluster of disease states that could be alleviated through eCB based medications.
